# Implementation of the Richmond Agitation-Sedation Scale (palliative version) on an inpatient palliative care unit

**DOI:** 10.1186/s12904-023-01298-y

**Published:** 2023-11-04

**Authors:** Shirley H. Bush, Katarzyna Bronicki, Michel Dionne, Natasha Lelievre, Peter G. Lawlor, Monisha Kabir

**Affiliations:** 1 Department of Medicine, Division of Palliative Care, University of Ottawa, Ottawa, ON Canada; 2 Bruyère Research Institute, Ottawa, ON Canada; 3 Clinical Epidemiology Program, Ottawa Hospital Research Institute, Ottawa, ON Canada; 4 Bruyère Continuing Care, Ottawa, ON Canada; 5 Department of Radiation Medicine, The Ottawa Hospital Cancer Centre, Ottawa, ON Canada

**Keywords:** Richmond Agitation-Sedation Scale (palliative version) – RASS-PAL, Implementation, Self-learning module, Palliative care, End-of-life care, Palliative sedation

## Abstract

**Background:**

The Richmond Agitation-Sedation Scale – Palliative version (RASS-PAL) tool is a brief observational tool to quantify a patient’s level of agitation or sedation. The objective of this study was to implement the RASS-PAL tool on an inpatient palliative care unit and evaluate the implementation process.

**Methods:**

Quality improvement implementation project using a short online RASS-PAL self-learning module and point-of-care tool. Participants were staff working on a 31-bed inpatient palliative care unit who completed the RASS-PAL self-learning module and online evaluation survey.

**Results:**

The self-learning module was completed by 49/50 (98%) of regular palliative care unit staff (nurses, physicians, allied health, and other palliative care unit staff). The completion rate of the self-learning module by both regular and casual palliative care unit staff was 63/77 (82%). The follow-up online evaluation survey was completed by 23/50 (46%) of respondents who regularly worked on the palliative care unit. Respondents agreed (14/26; 54%) or strongly agreed (10/26; 38%) that the self-learning module was implemented successfully, with 100% agreement that it was effective for their educational needs.

**Conclusion:**

Using an online self-learning module is an effective method to engage and educate interprofessional staff on the RASS-PAL tool as part of an implementation strategy.

**Supplementary Information:**

The online version contains supplementary material available at 10.1186/s12904-023-01298-y.

## Background

Pharmacological palliative sedation may be required to manage refractory distressing symptoms, such as agitated delirium, dyspnea, and pain, in the last days, up to two weeks of life [1, 2]. For best clinical practice, it is necessary to clinically monitor patients receiving palliative sedation [3–5]. This includes the use of validated tools to monitor a patient’s level of sedation such as the Richmond Agitation-Sedation Scale (RASS), which was developed and validated in intensive care unit patients, [6] and the palliative version, the RASS-PAL [7]. The RASS-PAL is a brief observational tool which was developed for an inpatient palliative care unit (PCU) and used by nurses and physicians to quantify a snapshot level of sedation or agitation in a patient. The observed RASS-PAL level can range from + 4 (combative) to -5 (not rousable) [7]. While previous studies have used the RASS and RASS-PAL to evaluate the level of agitation and sedation in patients to guide clinical care, to our knowledge there are no previously published detailed descriptions of RASS-PAL implementation for the healthcare team.

## Methods

### Aim and design of study

Following our recent implementation of a delirium guideline using self-learning modules (SLM), [8] there was an outstanding need to formally implement the RASS-PAL on our PCU. As part of the process of adaptation and implementation of a regional palliative sedation guideline, our project objective was to implement the RASS-PAL tool before it became part of standard electronic patient record (EPR) documentation as a quality improvement implementation project, and to evaluate the process.

The Standards for QUality Improvement Reporting Excellence (SQUIRE 2.0) reporting guideline was used to write up this project [9].

### Setting and context

This implementation project took place on a 31-bed inpatient PCU situated in a publicly funded subacute academic hospital in Ottawa, Canada. The unit has a total of 50 regular staff members. Registered Nurses and Registered Practical Nurses work 8-h shifts on the PCU. Rotating palliative care staff physicians work across the unit and provide a 24/7 physician on call roster. The other members of the PCU interprofessional team are: 1.0 FTE clinical manager, 0.5 FTE Nursing Practice Leader (NPL), 1.0 FTE Practice Support Nurse, 0.8 FTE social worker, 1.0 FTE pharmacist, and 0.5 FTE spiritual care provider. The PCU team is supported by a porter and ward clerks. As this is an academic teaching unit, medical students and medical residents are supervised during their 2 to 4-week clinical rotations. Our EPR system, MEDITECH (Medical Information Technology, Inc.), was introduced in January 2015. From April 2019—March 2020, there were 574 admissions. Posted admission criteria are available online [10].

### Process of RASS-PAL implementation using a self-learning module

Table 1 outlines the steps taken to implement the RASS-PAL tool. This included the completion of fishbone (Ishikawa) diagrams by the core project group to capture their thoughts surrounding barriers and facilitators to RASS-PAL implementation on the PCU. Our palliative care unit had used the Nursing Delirium Screening Scale (Nu-DESC) [11] for many years as a delirium screening tool at the end of each nursing shift. Thus, our RASS-PAL implementation strategy had to clearly contrast the rationale for the RASS-PAL with the ongoing use of the Nu-DESC to ensure staff understood the different indications for each tool. Initially our plan had been for RASS-PAL training to be specifically for nurses and physicians, but the core project group determined that we should develop an online self-learning module (SLM) which could be completed by *all* staff (including allied health, ward clerks and porter) due to the expectation that RASS-PAL levels would be routinely discussed during daily practice.

**Table 1:** Steps taken to implement Richmond Agitation-Sedation Scale—palliative version (RASS-PAL) using a self-learning module

(1) Key stakeholders were identified and recruited, including local EPR colleagues to facilitate addition of the RASS-PAL tool to the EPR. Once approved by EPR regional partners, the ‘go live’ date for the online version of the RASS-PAL tool was set for June 2019, aligning with an extensive preplanned EPR update
(2) A PCU interprofessional core project group was formed, comprising NPL (KB) and PSN, physicians (*n* = 2) (SB and MD), pharmacist, social worker, spiritual care provider, and clinical manager. The core project group defined a project timeline. In-person group meetings were held every 2 weeks from December 2018 to April 2019
(3) The core project group completed collaborative fishbone diagrams for major barriers and facilitators to implementation of the RASS-PAL on the PCU
(4) The project lead (SB) and NPL developed a short online interactive SLM for all staff. Content consisted of a clinical case of a patient with refractory agitated delirium at the end of life receiving palliative sedation. In addition to describing the RASS-PAL tool, this SLM contrasted the RASS-PAL with the Nursing Delirium Screening Scale (Nu-DESC).^11^ The SLM module ended with an interactive exercise and 4 mandatory post-module questions
(5) To assist implementation, a RASS-PAL point-of-care tool (as a double-sided ‘one-pager’ titled “Introducing the RASS-PAL”) for bedside nurses was developed by the NPL and a laminated copy was placed on every nurse mobile computer workstation (See Additional file 1).
(6) Laminated copies of the RASS-PAL tool and procedure were posted in all PCU-wings and the team rounds room as a reference document for all staff

The first part of the SLM described the RASS-PAL scale, explained the procedure for RASS-PAL assessment of a patient and how to determine a RASS-PAL level. It also provided a synopsis of the Nu-DESC to highlight the differences between the RASS-PAL and Nu-DESC tools, including their different roles and assessment timeframes. The second part of the SLM took the learner through a clinical case of a patient with agitated delirium who later required palliative sedation at the end of life, requiring the learner to determine the patient’s RASS-PAL level at each stage. Post-module questions were created for the final SLM which was uploaded onto the hospital online learning platform. The SLM was estimated to have a 10-min completion time.

### RASS-PAL SLM implementation

The clinical manager sent an email to all regular and casual PCU staff (*N* = 77) requesting completion of the SLM which had recently gone ‘live’ on March 7, 2019, and two reminder emails. For lock-in improvement post training to ensure that practice change was carried out on a regular basis, nursing staff were to enter a RASS-PAL level at least once every 8-h shift (at 02:00, 10:00 and 18:00) in the EPR for each patient as a minimum requirement. (Nurses are able to enter a RASS-PAL level more frequently if warranted, e.g. during the titration phase of palliative sedation medications). The NPL developed a RASS-PAL point-of-care tool (see Additional file 1) for the PCU nurses and posted laminated copies on the mobile computer workstations throughout the unit.

Twelve weeks post-implementation, a 50-min in person ‘reflections’ meeting was held with the core project group, facilitated and documented by the research assistant (MK). The following four questions were posed: (1) what worked well and why (2) what did not work well and why (3) what were the key learnings, and (4) what should be changed in the next improvement effort?

### Quality improvement measures

Our project process measure was for > 70% of the PCU interprofessional team to complete the SLM. We also aimed for > 30% completion rate of the evaluation survey. Outcome measures were team feedback in respect to the SLM being accessible and an effective mode for education. The impact of SLM completion time on staff workload was used as a balancing measure.

### Evaluation of implemented SLM

Online evaluation survey development was informed by the Theoretical Domains Framework [12–14] and the RE-AIM Framework [15]. Two interprofessional team members piloted the draft survey and minor revisions were made based on their feedback. Survey questions included participant demographics and concluded with an open-ended question for optional comments (Additional file 2). The clinical manager sent an email containing the ethics board approved invitation and survey link to all staff on March 27, 2019. The survey was administered through SurveyMonkey®. (SurveyMonkey: SurveyMonkey Inc. San Mateo, California, USA; 2019. www.surveymonkey.com). Two reminder emails were sent, and responses were collected until April 29, 2019.

### Analysis

Staff module completion was verified by the hospital education department. For all included survey responses, descriptive statistics were computed for the appropriate quantitative data using Microsoft Excel®. (Microsoft Excel, version 14.0: Microsoft Corporation, US; 2010). Individuals with incomplete responses were excluded from the analysis.

### Ethics approval

The project received approval from the local research ethics boards. Participants provided electronic consent to participate prior to completing the online evaluation survey.

## Results

### Fishbone exercise results

**Fig. 1:**
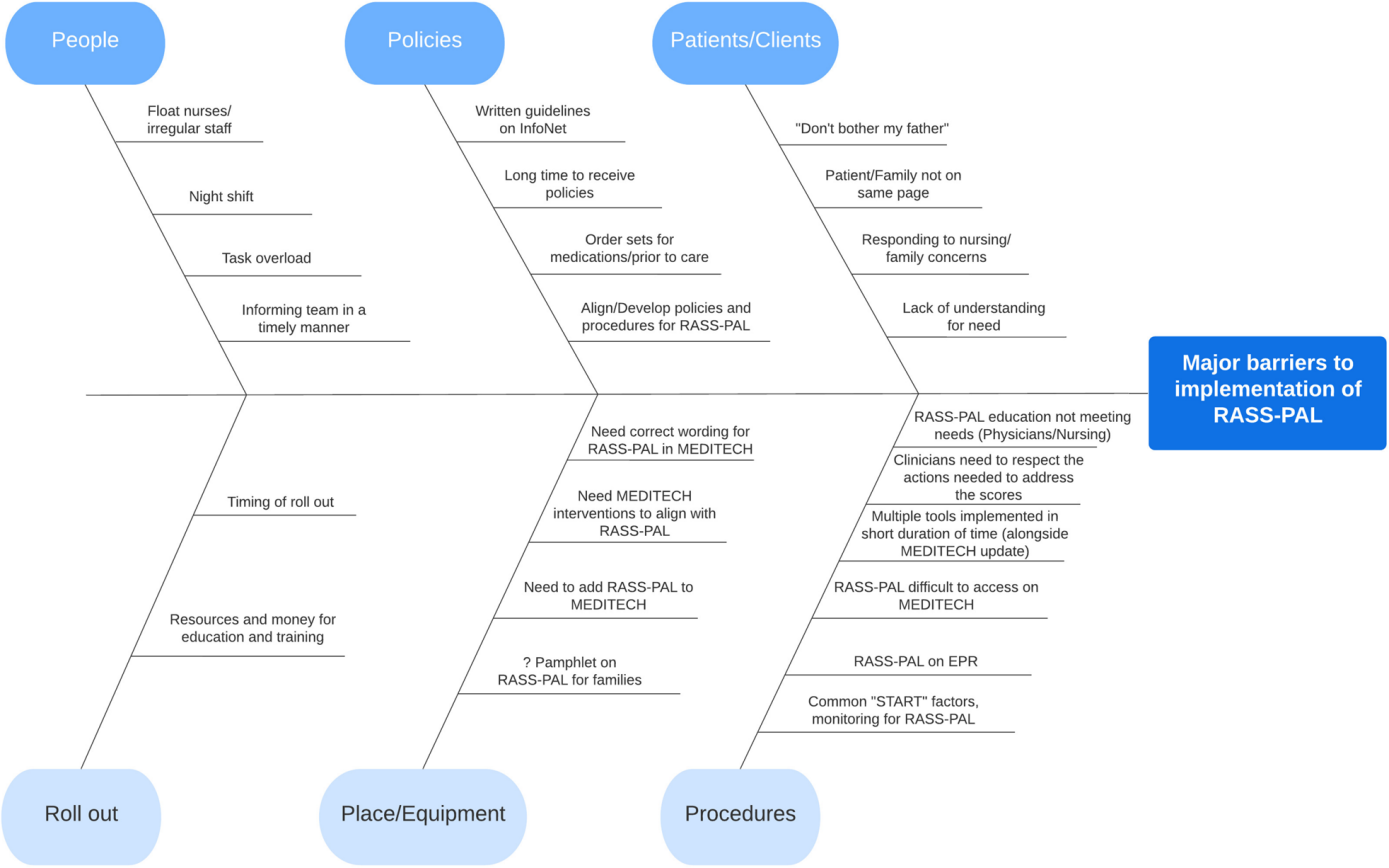
Major barriers to implementation of Richmond Agitation-Sedation Scale – Palliative version as identified by project group. Legend: InfoNet: internal website; MEDITECH: name of hospital electronic patient record, Medical Information Technology, Inc

**Fig. 2:**
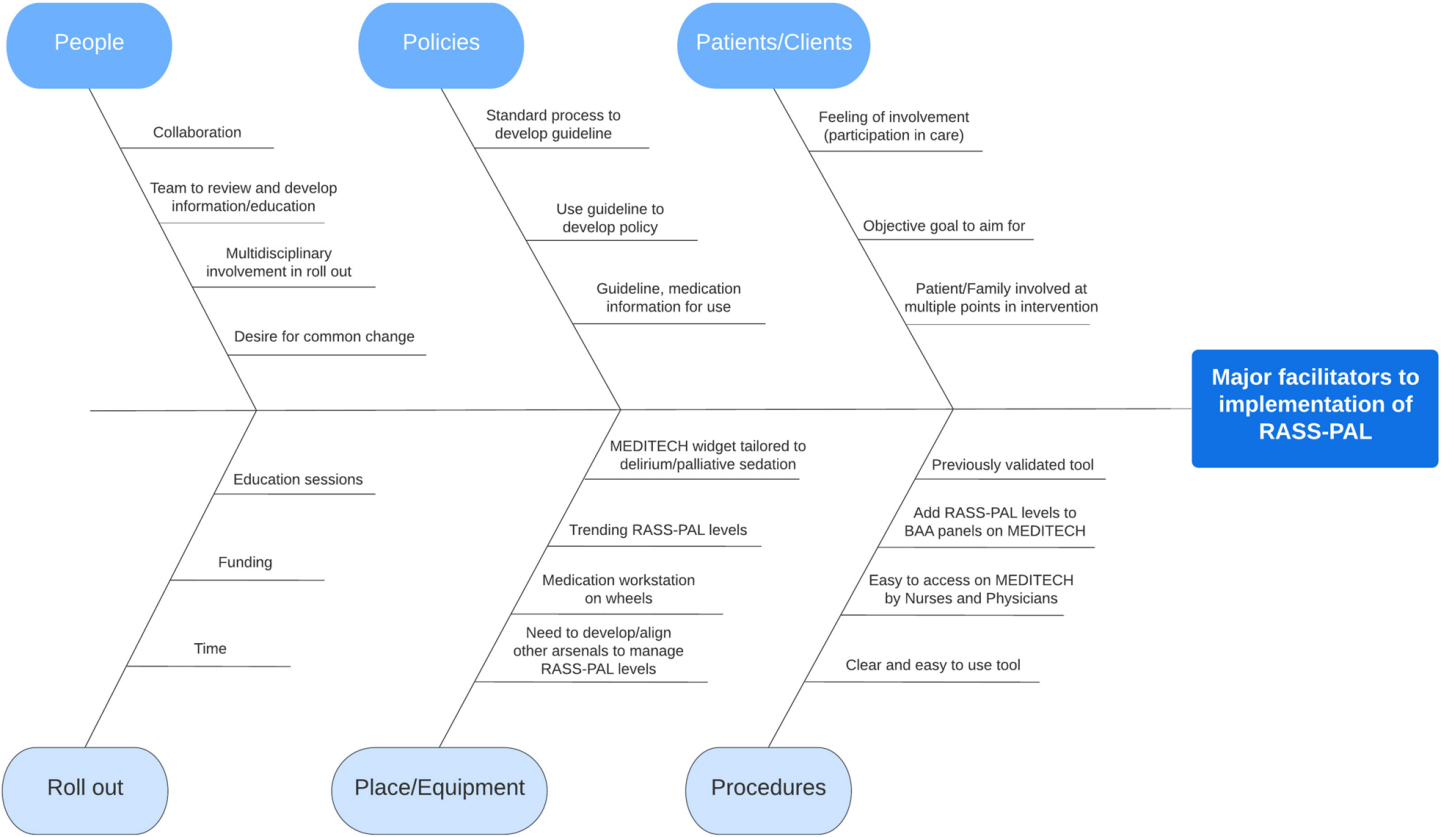
Major facilitators to implementation of Richmond Agitation-Sedation Scale – Palliative version as identified by project group. Legend: MEDITECH: name of hospital electronic patient record, Medical Information Technology, Inc

The main themes for major barriers to implementation of the RASS-PAL identified by the project group were: (1) the timing of rollout and reach to all PCU staff, (2) need for RASS-PAL specific education and resources, and (3) alignment with the EPR (Fig. 1). Regarding major facilitators, main themes were: (1) access of RASS-PAL tool on the EPR, and (2) need for collaboration in development of education and rollout (Fig. 2).

### RASS-PAL SLM completion

The overall SLM completion rate by regular and casual staff was 63/77 (82%). Nearly all (98%) of 50 regular PCU team members completed the SLM, with 100% completion rate by bedside nurses, senior nursing, physicians, and allied health (Table 2).

**Table 2:** Completion rates of RASS-PAL online self-learning module and evaluation survey by staff role

**PCU role**	**RASS-PAL Module** *N* (%)	**RASS-PAL Evaluation Survey** *N* (%)
Physician	9/9 (100)	6/9 (67)
Nurse (Regular and casual RN + RPN)	45/58 (78)	14/58 (24)
• Regular RN/RPN (PCU)	31/31 (100)	11/31 (35)
Senior Nursing (CM, NPL, PSN)	3/3 (100)	3/3 (100)
Allied Health (Pharmacist, social worker, spiritual care)	3/3 (100)	2/3 (67)
Other PCU staff (Ward clerks, porter)	3/4 (75)	1/4 (25)
**Total completion rate:**	**63/77 (82)**	**26/77 (34)**
• **Completion rate by regular PCU staff:**	**49/50 (98)**	**23/50 (46)**

### Evaluation survey

After excluding three incomplete evaluation survey responses, 26/77 (34%) responses from both regular and casual PCU staff were analyzed. A greater proportion of regular PCU staff, 23/50 (46%), completed the survey (Table 2). Respondent demographics are provided in Supplementary Table 1 in Additional file 3.

Survey respondents rated the SLM positively (Table 3). All survey respondents agreed that the SLM was an effective use of their time for education. Overall, respondents agreed (14/26; 54%) or strongly agreed (10/26; 38%) that the SLM on the RASS-PAL was implemented successfully and that it would impact their ongoing practice on the PCU (agreed: 14/26; 54%; strongly agreed: 12/26; 46%). The following are selected free-text comments:

- “[The SLM] provided a very clear and comprehensive summary of how to use RASS-PAL”
- “Quick and easy e-module for staff. Gets the message and key points across clearly. The use of several interactive prompts (e.g. quiz, tool box) I found helped to facilitate my engagement with the module”
- “Very helpful and with some adaptation could be very useful in other settings as well”

**Table 3:** Respondent evaluations of the RASS-PAL self-learning module based on evaluation survey responses (*N* = 26)

Question		Strongly Disagree		Disagree		Neutral		Agree		Strongly Agree		Weighted Average^a^ ± SD
Q8	The self-learning module on the RASS-PAL is accessible to those who are most in need of the knowledge	0	0%	0	0%	1	4%	12	46%	13	50%	4.46 ± 0.58
Q9	The self-learning module on the RASS-PAL is effective for my education needs on using the RASS-PAL	0	0%	0	0%	0	0%	10	38%	16	62%	4.62 ± 0.50
Q10	My use of the RASS-PAL has resulted in unintentional adverse outcomes	14	54%	6	23%	4	15%	2	8%	0	0%	1.77 ± 0.99
Q11	I feel comfortable in assessing patients with the RASS-PAL	0	0%	0	0%	2	8%	14	54%	10	38%	4.31 ± 0.62
Q12	My use of the self-learning module on the RASS-PAL has improved patient quality of care	0	0%	1	4%	8	31%	12	46%	5	19%	3.81 ± 0.80
Q13	The self-learning module on the RASS-PAL helps Bruyère Continuing Care achieve its mission, specifically: “the provision of evidence based health care and services for the vulnerable and medically complex, with a focus on persons who require sub-acute, geriatric or palliative care”	0	0%	0	0%	0	0%	17	65%	9	35%	4.35 ± 0.49
Q14	The self-learning module on the RASS-PAL was implemented successfully	0	0%	0	0%	2	8%	14	54%	10	38%	4.31 ± 0.62
Q15	The self-learning module on the RASS-PAL is an effective use of my time for education	0	0%	0	0%	0	0%	14	54%	12	46%	4.46 ± 0.51
Q16	The training I have received from the self-learning module on the RASS-PAL fits well with current routine practices on the PCU	2	8%	0	0%	1	4%	12	46%	11	42%	4.15 ± 1.08
Q17	The self-learning module on the RASS-PAL will impact my continuing practice on the PCU	0	0%	0	0%	0	0%	14	54%	12	46%	4.46 ± 0.51

^a^Based on ordinal values of 1, 2, 3, 4, 5 assigned to the categories ‘Strongly Disagree’, ‘Disagree’, ‘Neutral’, ‘Agree’, ‘Strongly Agree’, respectively, wherein Strongly Disagree = 1, Disagree = 2, Neutral = 2, Agree = 4, Strongly Agree = 5

### Lock-in improvement

A PCU nursing self-audit conducted seven months post RASS-PAL implementation reported 100% compliance with RASS-PAL documentation in the EPR for each shift.

### Core project group reflections

Five core project group members attended the ‘reflections’ meeting. Collaboration and engagement with the PCU team was highlighted, with the importance of the NPL contribution to ensure that the clinical case in the SLM resonated with nurses. The project also demonstrated the utility of RASS-PAL implementation to clinical care. Targeting the whole PCU team for SLM completion was felt to be an important means of increasing everyone’s awareness of critical situations to be reported to a patient’s nurse and physician. The group’s previous delirium guideline implementation work [8] had built confidence and made the RASS-PAL implementation feel “more smooth, engaging, and more team-focused”. Project challenges included NPL turnover, and time restraints for attending project group meetings due to other priorities. Key learnings were that the inclusion of more interprofessional members in this project group allowed for different perspectives and input which should be continued in future quality improvement initiatives. Using technology, such as teleconferencing or video conferencing, was recommended to increase project team meeting attendance and engagement.

## Discussion

This work advances evidence-based practice by addressing a crucial aspect of patient care – the assessment of agitation and sedation – within the context of an inpatient palliative care unit. Our report describes in detail the steps taken to implement the RASS-PAL tool into clinical practice on an inpatient palliative care unit before it is used for patient assessment by the clinical team, utilizing a standardized approach, active engagement of key stakeholders, and well-structured self-learning module as a crucial education intervention for all staff working on the palliative care team, not just nurses and physicians.

In this project, we found that the new SLM was an effective education modality to reach core staff and successfully implement the RASS-PAL tool on our inpatient PCU. There was a high online SLM completion rate with staff agreeing that the education initiative was effective. The engagement of EPR stakeholders at the outset of this project was critical in facilitating the addition of the RASS-PAL tool as part of a planned significant EPR update as a priority item, thus avoiding a potential barrier. The final timing of SLM rollout was selected to avoid a period of multiple annual leave absences, thereby increasing the reach of the RASS-PAL education to regular PCU staff. A unique feature of this project was the requirement for the SLM to be completed by all staff, irrespective of their role on the PCU. Similar to reports from other implementation projects, challenges occurred with changes in personnel (NPL) during the project, limited staff time, and a need to adjust the project timeline due to other competing internal and external projects [16–18].

An important lesson learned during the project was that, in contrast to our initial RASS-PAL validation study, [7] the instructions for assessment required modification so that the clinical team incorporated their RASS-PAL assessment as part of routine care (e.g. when giving medications, hygiene, or with turns) and did not purposefully wake any patient who was receiving palliative sedation. This new recommendation was included in the SLM.

To sustain implementation efforts, a briefer ‘refresher’ SLM (to cover just the RASS-PAL tool) was created to accompany the future implementation of a palliative sedation guideline. A version of this ‘refresher’ SLM was also created for external providers with online access provided via our hospital website [19]. Information on the RASS-PAL tool was added to the medical learner orientation package. We also added “RASS-PAL target” as an option for physicians to complete on our midazolam infusion order set. While the midazolam infusion order set is currently paper based, an accompanying dedicated entry field for “RASS-PAL target” was created within the EPR.

### Strengths and limitations

A project strength was the use of an interprofessional project group from inception to post-implementation evaluation. A limitation is the 46% response rate to the evaluation survey by regular PCU staff. Future survey completion rates may be augmented by providing frontline staff with dedicated time for such tasks. Limitations also include the need for further RASS-PAL validation studies in other centers, in addition to comparative studies with other bedside observational tools [4]. For patients receiving continuous palliative sedation, a measure of patient comfort should also be included [20].

## Conclusions

The detailed description of the methods utilized for RASS-PAL implementation, and sharing of key resources, provide a practical guide for replicating similar quality improvement/ implementation initiatives in other settings. An online SLM can be used to engage and educate all staff members for RASS-PAL implementation. Further RASS-PAL validation and implementation studies are needed.

### Supplementary Information

**Additional file 1.** Point-of-care Tool: Introducing the RASS-PAL.

**Additional file 2.** Evaluation Survey: The RASS-PAL (Palliative version of the Richmond Agitation-Sedation Scale) Self-Learning Module.

**Additional file 3:** **Supplementary Table 1.** Demographics of evaluation survey respondents (*N *=26).

## Data Availability

The study data are held at the Bruyère Research Institute. The datasets used and analyzed during the current study are available from the corresponding author on reasonable request.

## Abbreviations

CM: Clinical manager
EPR: Electronic patient record
NPL: Nursing Practice Leader
Nu-DESC: Nursing Delirium Screening Scale
PCU: Palliative care unit
PSN: Practice Support Nurse
RASS: Richmond Agitation-Sedation Scale
RASS-PAL: Richmond Agitation-Sedation Scale – Palliative version
RN: Registered Nurse
RPN: Registered Practical Nurse
SLM: Self-learning module
SQUIRE: Standards for Quality Improvement Reporting Excellence
