# Supplementary material for: Implementation of the Richmond Agitation-Sedation Scale (palliative version) on an inpatient palliative care unit
Source: BMC Palliat Care. 2023 Nov 4;22:171. doi: 10.1186/s12904-023-01298-y (PMC10625230; doi:10.1186/s12904-023-01298-y)
Supplement: Supplementary file 1 — Additional file 1. Point-of-care Tool: Introducing the RASS-PAL. [file 12904_2023_1298_MOESM1_ESM.pdf]

Point-of-care Tool: Introducing the RASS-PAL

What is it? The **RASS** (Richmond Agitation-Sedation Scale) is a simple **observational** tool initially developed for ICU nurses to assess their patients. The RASS was modified for patients on a palliative care unit and the **RASS-PAL** was created. The tool gives a snapshot of a patient's condition during **a 20 second period of assessment.**

Why is this important? It helps to assess a patient's degree of **agitation** or **sedation.** The **RASS-PAL** provides a way to **communicate** clinical findings in a **standardized** way in daily practice.

Who uses it? RASS-PAL will be used by **nurses** and **physicians.**

What does RASS-PAL look like in the EPR?

by KH

Interventions
##PALL-Richmond Agitation Sedation Scale ✓
Assessments
PALL-Richmond Agitation and Sedation Scale (Palliative Version) ✓
RASS-PAL
Richmond Agitation and Sedation Scale (RASS-PAL)

- ○ +4 combative
  overtly combative, violent, immediate danger to staff (e.g.throwing items)
  + / - attempting to get out of bed or chair
- ○ +3 very agitated
  pulls or remove lines (e.g. IV/ SQ/ oxygen tubing) or catheter(s); aggressive
  + / - attempting to get out of bed or chair
- ○ +2 agitated
  frequent non-purposeful movement, +/ - attempting to get out of bed or chair
- ○ +1 restless
  occasional non-purposeful movement, but movements not aggressive or vigorous
- ○ 0 alert and calm
- ○ -1 drowsy
  not fully alert, but has sustained awakening (eye opening/ eye contact) to voice (10 seconds or longer)
- ○ -2 light sedation
  briefly awakens with eye contact to voice (for less than 10 seconds)
- ○ -3 moderate sedation
  any movement (eye or body) or eye opening to voice (but no eye contact)
- ○ -4 deep sedation
  no response to voice, but any movement (eye or body) or eye opening to stimulation by light touch
- ○ -5 not rousable
  no response to voice or to stimulation by light touch

How is it used? Using your 20 second assessment, follow these instructions to rate your patient. DO NOT intentionally WAKE your patient to assess RASS-PAL levels – do it as part of your ongoing care and monitoring.

PROCEDURE FOR RASS-PAL ASSESSMENT

1. Observe patient for **20 seconds.**	
a. Patient is alert, restless, or agitated **for more than 10 seconds**	**Score 0 to +4**
**Note:** If patient is alert, restless, or agitated for less than 10 seconds and is otherwise drowsy, then score patient according to your assessment for the majority of the observation period	
2. If not alert, greet patient and call patient by name and *say* to open eyes and look at speaker.	
b. Patient awakens with sustained eye opening and eye contact **(10 seconds or longer).**	**Score -1**
c. Patient awakens with eye opening and eye contact, but not sustained **(less than 10 seconds).**	**Score -2**
d. Patient has any eye or body movement in response to voice but no eye contact.	**Score -3**
3. When no response to verbal stimulation, physically stimulate patient by light touch e.g. gently shake shoulder.	
e. Patient has any eye or body movement to gentle physical stimulation.	**Score -4**
f. Patient has no response to any stimulation.	**Score -5**

These 3 images are from:

Bush SH et al. BMC Palliat Care 2014 Mar 31; 13(1):17.

See end of document for full citation

AGITATION

Score	Term	Description
+4	Combative	Overtly combative, violent, immediate danger to staff (e.g. throwing items); +/- attempting to get out of bed or chair
+3	Very agitated	Pulls or removes lines (e.g. IV/SQ/Oxygen tubing) or catheter(s); aggressive, +/- attempting to get out of bed or chair
+2	Agitated	Frequent non-purposeful movement, +/- attempting to get out of bed or chair
+1	Restless	Occasional non-purposeful movement, but movements not aggressive or vigorous

SEDATION

0	Alert and calm		
-1	Drowsy	Not fully alert, but has sustained awakening (eye-opening/eye contact) to *voice* **(10 seconds or longer)**	Verbal Stimulation
-2	Light sedation	Briefly awakens with eye contact to *voice* **(less than 10 seconds)**	Verbal Stimulation
-3	Moderate sedation	Any movement (eye or body) or eye opening to *voice* **(but no eye contact)**	Verbal Stimulation
-4	Deep sedation	No response to voice , but any movement (eye or body) or eye opening to *stimulation by light touch*	Gentle Physical Stimulation
-5	Not rousable	No response to *voice or stimulation by light touch*	Gentle Physical Stimulation

What does the tool tell me? A patient's level of agitation or sedation at a **particular moment in time**. For example, it can help you to assess the effectiveness of antipsychotic or benzodiazepine medications. It can also help with the assessment of patients receiving Palliative Sedation and assist with the adjustments of these medications.

What do I do with this information? Your assessment is documented in **Meditech**. Communicate your **RASS-PAL** rating to your nursing colleagues at handover and bedside shift report, and with physicians.

How often should I assess my patient with RASS-PAL? The RASS-PAL should be done on admission and at least once per shift (at the beginning of your shift). Do the RASS-PAL more often depending on the clinical situation, such as before and after medication for agitation. Attach a note describing your assessments and interventions.

Point-of-care Tool: RASS-PAL Communication and Documentation

1. Document in Clinical Data (under Patient Info: Alerts) that patient has been initiated on Palliative Sedation.
2. Under the MDS Level of Consciousness, (Alert, Somnolent, and Obtunded/Comatose), translate to the following RASS-PAL level: MDS Alert = RASS-PAL 0, MDS Somnolent = RASS-PAL -1 to -4, MDS Obtunded/Comatose = RASS-PAL -5
3. Add a **SOAP** note under MDS Level of Consciousness. For the title, type "RASS-PAL Assessment for Palliative Sedation".
4. **Subjective Data:** Add something if shared by patient or family and appropriate (Note: patient may ultimately not be able to share subjective concerns if the intent is for a deep level of sedation).
5. **Objective Data:** Enter your RASS-PAL level 0 to -5 along with the Term for the level (this comes right off the RASS-PAL Sedation Scale).
6. **Assessment**: Enter the Description noted from your assessment, AGAIN using the words noted on the RASS-PAL Sedation Scale. Document any other findings you note for your patient.
7. **Plan**: If patient is on a CADD for Palliative Sedation purposes, document your CADD settings and ALL "Clinician boluses" and the infusion rate to achieve the targeted RASS-PAL level. Also document if you have to page, call and speak with the physician for additional interventions to help optimize the level of Palliative Sedation for your patient.

*COMMUNICATE the current RASS-PAL sedation level and goal for sedation (target RASS-PAL level) with each and every Bedside Shift Report – ensure that your incoming colleague understands the tool and documentation.

Report and Documentation Examples

Patient <u>without</u> Palliative Sedation

*You are the **day** nurse providing report to the **evening** nurse caring for Mr. Jones who has been agitated. Your report on the RASS-PAL might be as follows:*

At 1330, Mr. Jones was agitated due to his delirium, RASS-PAL was +3, he pulled out his IV line and was pulling at his foley catheter. I talked to Dr. Brown and I gave Mr. Jones Haldol 0.5mg Subcut and Midazolam 1mg Subcut. He looks more comfortable and settled now, his RASS-PAL is +1 and his Nu-DESC for DAY shift was 5. The bed alarm is on for safety. He is a high fall risk. His son is usually at the bedside and he is aware of the fall risk**.**

*SOAP note example for an agitated patient **WITHOUT** Palliative Sedation:*

Subjective: Pt asking writer **"**What time is it? What do I do? I'm so confused. Can I go home?"

Objective: At 1330, pt. calling out, audible in hallway. Pt pulled out IV and pulling at foley catheter. Emotional support and reorientation provided and comforting family presence encouraged. PRN Haldol 0.5mg Subcut and Midazolam 1mg Subcut given. Bed in lowest position with 3 rails up. Bed alarm on. RASS-PAL +3, very agitated.

Assessment: Very agitated

Plan: Dr. Brown in assessing patient. Ongoing follow-up by writer.

Patient <u>with</u> Palliative Sedation

*You are the **evening** nurse providing care to Mr. Jones. A family meeting has taken place and **Palliative Sedation** is to be initiated with Midazolam Subcut CADD infusion. Concentration: 2mg/ml, Infusion Rate: 1mg/hr, PCA Bolus dosage: N/A. Clinician bolus: 1mg q30min PRN. Call MD if more than 3 clinician boluses required per nursing shift.*

*Your **SOAP note** at the start of shift might be as follows:*

Subjective: POA (patient's son) has discussed with team regarding starting palliative sedation to ease patient's distress due to agitated delirium.

Objective: Baseline RASS-PAL level +3, very agitated

Assessment: Patient very agitated. Son at bedside with patient. Both patient and son prepared for initiation of CADD Midazolam. All orders reviewed.

Plan: CADD Subcut Midazolam 2mg/ml initiated at 15:30. Clinician bolus of Midazolam 1mg Subcut given and CADD infusing at 1mg/hour with goal to reach target level of RASS-PAL -4.

**Please refer to binder located at nursing station B, D, and E for Meditech examples and further RASS-PAL information.

Source document for RASS-PAL: Bush SH, Grassau PA, Yarmo MN, Zhang T, Zinkie SJ, Pereira JL. The Richmond Agitation-Sedation Scale modified for palliative care inpatients (RASS-PAL): A pilot study exploring validity and feasibility in clinical practice. BMC Palliat Care 2014 Mar 31; 13(1):17. doi: 10.1186/1472-684X-13-17.
