# Supplementary material for: Implementation of the Richmond Agitation-Sedation Scale (palliative version) on an inpatient palliative care unit
Source: BMC Palliat Care. 2023 Nov 4;22:171. doi: 10.1186/s12904-023-01298-y (PMC10625230; doi:10.1186/s12904-023-01298-y)
Supplement: Supplementary file 2 — Additional file 2. Evaluation Survey: The RASS-PAL (Palliative version of the Richmond Agitation-Sedation Scale) Self-Learning Module. [file 12904_2023_1298_MOESM2_ESM.pdf]

Evaluation Survey: The RASS-PAL (Palliative version of the Richmond Agitation-Sedation Sca Self-Learning Module

PARTICIPANT INFORMED CONSENT FORM

Online Survey Evaluating RASS-PAL Self-Learning Module

Title of Study: Palliative Sedation (PS) guideline implementation: The development of online education modules for staff, and a patient and family information leaflet

Local Site Principal Investigator (PI): Dr. Shirley H. Bush, 613-562-6262, Ext 1060

Funder: Bruyère Academic Medical Organization (BAMO)

Participation in this study is voluntary. Please read this Participant Informed Consent Form carefully before you decide if you would like to participate. Ask the study team as many questions as you like.

Why am I being given this form?

You are being asked to participate in this research study because you are a clinical team member at the Élisabeth Bruyère Hospital (EBH), on the Palliative Care Unit (PCU).

Why is this study being done?

Patients in the last days of life (up to 2 weeks of life) may require sedation to manage refractory distressing symptoms, such as agitated delirium, dyspnea, and uncontrolled pain. This is also known as palliative sedation (PS) or sedation in the terminal phase.

The Principal Investigator (S. Bush) has recently completed the implementation of a Delirium Clinical Practice Guideline (CPG) for palliative care at Bruyère. An online education module on PS is now needed for the PCU interprofessional (IP) team to complement this work. One of the Co-Investigators (J. Rice) has been leading the development of a new PS CPG for the Champlain LHIN. Our research team will adapt this to replace the current PS CPG on the palliative care unit (PCU) at Élisabeth Bruyère Hospital. Also critical to the implementation of this PS CPG are: education on the Richmond Agitation-Sedation Scale – palliative version (RASS-PAL) to monitor PS, and the provision of information and support to patients and their families via an information leaflet on PS.

How is the study designed?

The research team will review the new Champlain LHIN PS CPG and modify it (if needed) for the Bruyère PCU context.

The researchers will also develop, pilot-test, and finalize two self-learning modules (SLMs) for the PCU team based on a comprehensive scoping review. The SLMs will incorporate clinical cases which simulate clinical practice and pre-post tests for both the PS CPG and the implementation of the RASS-PAL tool.

What is expected of me?

You will be asked to participate in one (1) online survey on the Survey Monkey platform which asks you about how the PS CPG was developed and implemented on the PCU. The survey will take approximately 5-10 minutes to complete. You may skip any questions that make you uncomfortable or that you do not wish to answer.

How long will I be involved in the study?

The entire study will last approximately one year. Your participation in the on-line survey part of the study will be one-time only. Completion of the on-line survey will be done outside of paid work hours.

What are the potential risks I may experience?

You might not like all of the questions that you are asked. You do not have to answer any questions that make you uncomfortable.

Can I expect to benefit from participating in this research study?

You will not receive any direct benefit from your participation in this study. Your participation may allow the researchers to

<u>How is my personal information being protected?</u>

All information collected during your participation in this survey will be anonymous. Information that identifies you will be released only if it is required by law.

For audit purposes only, your original study records may be reviewed under the supervision of Dr. Shirley H. Bush's staff by representatives from: the Ottawa Health Science Network Research Ethics Board (OHSN-REB), the Bruyère Continuing Care Research Ethics Board (BCC-REB), Bruyère Continuing Care, and the Ottawa Hospital Research Institute. Research records will be kept for 10 years, after this time they will be destroyed.

<u>Who do I contact if I have any further questions?</u>

If you have any questions about this study, please contact Dr. Shirley H. Bush, Principal Investigator at 613-562-6262 Ext 1060 or the study staff at 613-562-6262 Ext 1590.

The OHSN-REB and the BCC-REB have reviewed the plans for this research study. Each REB considers the ethical aspects of all research studies involving human participants at Bruyère Continuing Care. If you have any questions about your rights as a study participant, you may contact the OHSN-REB Chairperson at 613-798-5555, extension 16719, or the Bruyère Continuing Care Research Ethics Board at 613-562-6262, extension 4003. Completion of the survey implies your consent to participate in this research study.

Consent form on following page

Evaluation Survey: The RASS-PAL (Palliative version of the Richmond Agitation-Sedation Sca
Self-Learning Module

PARTICIPANT INFORMED CONSENT FORM

Online Survey Evaluating RASS-PAL Self-Learning Module

Consent to Participate in Research:

I understand that I am being asked to participate in a research study about the implementation of a palliative sedation clinical practice guideline on the Palliative Care Unit. I have read each page of this Participant Informed Consent Form. All of my questions have been answered to my satisfaction. If I decide later that I would like to withdraw my participation and/or consent from the study, I can do so at any time. I voluntarily agree to participate in this study. I can save or print a copy of this signed Participant Informed Consent Form for my documents.

1. **Authorization:** In checking the box below **I** voluntarily agree to participate in this study.

◯ I agree

PARTICIPANT INFORMED CONSENT FORM

Online Survey Evaluating RASS-PAL Self-Learning Module

* 2. **<u>You have selected that you would like to participate in this study</u>.**
Please select <u>Yes</u> should you want to proceed. If you do not want to participate, please select <u>No</u>.

- ◯ Yes
- ◯ No

<u>Investigator Statement</u>

The nature of the above research study has been carefully outlined to the research participant. To the best of my knowledge, the research participant understands the nature, demands, risks and benefits involved in participating in this study. I acknowledge my responsibility for the care and well being of the above research participant, to respect the rights and wishes of the research participant, and to conduct the study according to applicable Good Clinical Practice guidelines and regulations.

Shirley H. Bush, MBBS, DRCOG, DCH, MRCGP, Dip Pall Med, FAChPM
Assistant Professor, Division of Palliative Care, University of Ottawa
Palliative Care Physician, The Ottawa Hospital / Bruyère Continuing Care
Clinical Scientist, Bruyère Research Institute (BRI)
43 Bruyère Street, Ottawa, ON K1N 5C8
Tel: 613-562-6262 ext 1060 Fax: 613-562-6371
sbush@bruyere.org

Evaluation Survey: The RASS-PAL (Palliative version of the Richmond Agitation-Sedation Sca
Self-Learning Module

Section I: Demographics

* 3. What is your role on the Palliative Care Unit (PCU)?

- PCU Physician
- Resident or Fellow
- PCU Pharmacist
- Registered Nurse (RN)
- Registered Practical Nurse (RPN)
- Advanced Practice Registered Nurse (APN)
- Social Worker
- Spiritual Care Worker
- Other, please specify...

* 4. How long have you worked on the Elisabeth Bruyère PCU?

- 0-5 years
- 6-10 years
- 11-15 years
- >15 years

Please specify total number of years worked in palliative care (ie. at Bruyère and elsewhere)

* 5. What is your employment status on the PCU?

- Full-time regular PCU staff
- Part-time regular PCU staff
- Non-regular PCU staff
- Other, please specify...

* 6. What shift do you primarily work?

- ◯ Day
- ◯ Evening
- ◯ Night
- ◯ Other, please specify...

____

* 7. Did you complete the RASS-PAL (Palliative version of the Richmond Agitation-Sedation Scale) self-learning module?

- ◯ Yes
- ◯ No

If no, please provide reason(s):

____

Evaluation Survey: The RASS-PAL (Palliative version of the Richmond Agitation-Sedation Sca Self-Learning Module

Section II: The RASS-PAL (Palliative version of the Richmond Agitation-Sedation Scale) Self-Learning

The following are scale-based questions. Please check one box for each question.

1=Strongly Disagree..........2=Disagree..........3=Neutral..........4=Agree..........5=Strongly Agree

Note: Some questions may not be relevant to all team members. If so, there is a 'Not applicable' option.

* 8. The self-learning module on the RASS-PAL is accessible to those who are most in need of the knowledge.

Strongly Disagree	Disagree	Neutral	Agree	Strongly Agree
◯	◯	◯	◯	◯

* 9. The self-learning module on the RASS-PAL is effective for my education needs on using the RASS-PAL.

Strongly Disagree	Disagree	Neutral	Agree	Strongly Agree
◯	◯	◯	◯	◯

* 10. My use of the RASS-PAL has resulted in unintentional adverse outcomes.

Strongly Disagree	Disagree	Neutral	Agree	Strongly Agree
◯	◯	◯	◯	◯

Please describe:

Section II: The RASS-PAL (Palliative version of the Richmond Agitation-Sedation Scale)

The following are scale-based questions. Please check one box for each question.

1=Strongly Disagree..........2=Disagree..........3=Neutral..........4=Agree..........5=Strongly Agree

Note: Some questions may not be relevant to all team members. If so, there is a 'Not applicable' option.

* 11. I feel comfortable in assessing patients with the RASS-PAL.

Strongly Disagree	Disagree	Neutral	Agree	Strongly Agree
○	○	○	○	○

* 12. My use of the self-learning module on the RASS-PAL has improved patient quality of care.

Strongly Disagree	Disagree	Neutral	Agree	Strongly Agree
○	○	○	○	○

Please describe:

* 13. The self-learning module on the RASS-PAL helps Bruyère Continuing Care achieve its mission, specifically: "the provision of evidence based health care and services for the vulnerable and medically complex, with a focus on persons who require sub-acute, geriatric or palliative care".

Strongly Disagree	Disagree	Neutral	Agree	Strongly Agree
○	○	○	○	○

Evaluation Survey: The RASS-PAL (Palliative version of the Richmond Agitation-Sedation Sca
Self-Learning Module

Section II: The RASS-PAL (Palliative version of the Richmond Agitation-Sedation Scale) Self-Learning

The following are scale-based questions. Please check one box for each question.

1=Strongly Disagree..........2=Disagree..........3=Neutral..........4=Agree..........5=Strongly Agree

Note: Some questions may not be relevant to all team members. If so, there is a 'Not applicable' option.

* 14. The self-learning module on the RASS-PAL was implemented successfully.

Strongly Disagree	Disagree	Neutral	Agree	Strongly Agree
○	○	○	○	○

* 15. The self-learning module on the RASS-PAL is an effective use of my time for education.

Strongly Disagree	Disagree	Neutral	Agree	Strongly Agree
○	○	○	○	○

* 16. The training I have received from the self-learning module on the RASS-PAL fits well with current routine practices on the PCU.

Strongly Disagree	Disagree	Neutral	Agree	Strongly Agree
○	○	○	○	○

* 17. The self-learning module on the RASS-PAL will impact my continuing practice on the PCU.

Strongly Disagree	Disagree	Neutral	Agree	Strongly Agree
○	○	○	○	○

Evaluation Survey: The RASS-PAL (Palliative version of the Richmond Agitation-Sedation Sca Self-Learning Module

Section III: Comments

The following is an open-ended question.

18. Do you have any further comments or concerns surrounding the RASS-PAL self-learning module?

Please note that once you click 'Done', the data used from this survey will no longer be able to be withdrawn from the study.

Thank you for your time in answering this survey.
