# Supplementary material for: Implementation of the Richmond Agitation-Sedation Scale (palliative version) on an inpatient palliative care unit
Source: BMC Palliat Care. 2023 Nov 4;22:171. doi: 10.1186/s12904-023-01298-y (PMC10625230; doi:10.1186/s12904-023-01298-y)
Supplement: Supplementary file 3 — Additional file 3: Supplementary Table 1. Demographics of evaluation survey respondents (N =26). [file 12904_2023_1298_MOESM3_ESM.docx]

Supplementary Table 1. Demographics of evaluation survey respondents (*N*=26)

| **Characteristics** | **Subcategories** | ***N* (%)** |
| --- | --- | --- |
| Role on PCU | Physician | 6 (23) |
|  | Nurse (RN, RPN) | 14 (54) |
|  | Senior Nursing (CM, NPL, PSN) | 3 (12) |
|  | Allied Health (Pharmacist, social worker, spiritual care) | 2 (8) |
|  | Other PCU staff (Ward clerks, porters) | 1 (4) |
| Length of time working on the Bruyère PCU | 0-5 years | 16 (62) |
|  | 6-10 years | 4 (15) |
|  | 11-15 years | 3 (12) |
|  | >15 years | 3 (12) |
| Employment status | Full-time regular PCU staff | 13 (50) |
|  | Part-time regular PCU staff | 9 (35) |
|  | Non-regular PCU staff | 4 (15) |
| Primary shift worked | Day | 18 (69) |
|  | Evening | 4 (15) |
|  | Night | 3 (12) |
|  | On call | 1 (4) |

**Abbreviations:**

CM: clinical manager

NPL: Nursing Practice Leader

PCU: palliative care unit

PSN: Practice Support Nurse

RN: Registered Nurse

RPN: Registered Practical Nurse
